# Supplementary material for: Retrospective record review of pregnant women treated for rifampicin-resistant tuberculosis in South Africa
Source: PLoS One. 2020 Sep 24;15(9):e0239018. doi: 10.1371/journal.pone.0239018 (PMC7514075; doi:10.1371/journal.pone.0239018)
Supplement: Attachment — Submitted filename: Response to Reviewers Comments.docx [file pone.0239018.s002.docx]

**Response to Reviewers Comments**

Reviewer #1: Walt et al, nicely reported on pregnancy and treatment outcomes of pregnant women with DR-TB among South-Africa population:

Following suggestive comments would make the manuscript stronger and beneficial for the readers:
Abstract:

• Word limits excided as per Journal guidelines. Should be modified.

**Response: Abstract revised to 300 words**

• While considering the word limit, author should write about the total number of patients screened and enrolled in study.

**Response: The facilities were requested to identify the records for women who were or became pregnant during DR-TB treatment. A total 26 records identified by the facility were reviewed. (See line 76)**

Method:

• If possible, author should specify the type of TB infection (PTB or EPTB) among patients.

**Response: Records of women who were pregnant before or while receiving individualized DR-TB therapy, with pulmonary tuberculosis disease between January 2010 and December 2018 were reviewed. (See line 70-71)**

• Authors should present sociodemographic data as maintained the method section.

**Response: Sentence corrected. We collected sociodemographic age and clinical information, including TB history and outcomes, drug-resistance, regimens, ADEs, treatment outcomes and birth outcome. (See line 79-80)**

Table 1: Age group, Years; 15-20. Author should remove the age limit because of no patients were enrolled in this group.

**Response: Age group removed. (See Table 1, line 108)**

Reviewer #2: This retrospective study provides information regarding Anti tuberculous treatment outcome on pregnant women. However, as the data suggests HIV positive percentage among the cohort was high, its correlation with the study outcome on successful birth is not briefed in the present manuscript. This point needs to be highlighted in the revised manuscript.

**Response: There was no statistically significant association between the mother’s HIV status and the outcome of the pregnancy (data not shown). Ultimately, whether the mother was tested positive or negative for HIV did not affect the outcome for the fetus. (See line 117-120)**

6. PLOS authors have the option to publish the peer review history of their article ([what does this mean?](https://protect-za.mimecast.com/s/wkVlCxGzZ5FJvYGzCAxlCv)). If published, this will include your full peer review and any attached files.
